# Self-Reported and Tested Function in Health Care Workers with Musculoskeletal Disorders on Full, Partial or Not on Sick Leave

**DOI:** 10.1007/s10926-014-9557-y

**Published:** 2014-11-27

**Authors:** Tove Ask, Jan Sture Skouen, Jörg Assmus, Alice Kvåle

**Affiliations:** 1 Physiotherapy Research Group, Department of Global Public Health and Primary Care, University of Bergen, PO Box 7800, 5020 Bergen, Norway; 2 The Outpatient Spine Clinic, Department of Physical Medicine and Rehabilitation, Haukeland University Hospital, Bergen, Norway; 3 Centre for Clinical Research, Haukeland University Hospital, Bergen, Norway

**Keywords:** Musculoskeletal pain, Sick leave, Disability, Work

## Abstract

**Purpose:**

The aim of this study was to describe self-reported and physically tested function in health care workers with musculoskeletal disorders (MSDs) and to examine how function was associated with work participation.

**Methods:**

A cross-sectional study was conducted. 250 health care workers attended an evaluation where self-reported and physical function were measured. Differences between groups (full sick leave, partial sick leave, not on sick leave/working) were analyzed for categorical data (Chi square exact test) and continuous variables (Kruskal–Wallis and Mann–Whitney U tests). Logistic regression analysis was performed to examine which factors were associated with being on sick leave.

**Results:**

Participants on full sick leave had statistically significant poorer function compared to those working and the group on partial sick leave. Logistic regression showed that a reduced level of the physical dimension of SF-12 and a high lift test were significantly related to full sick leave (OR 0.86, *p* < 0.001) (OR 0.79, *p* = 0.002). The physical dimension of SF-12 was the only variable that was associated to partial sick leave (OR 0.91, *p* = 0.005).

**Conclusion:**

Health care workers on full sick leave due to MSDs have reduced function on self-reported and physically tested function, compared to those working despite MSDs, as well as when compared to those on partial sick leave. More knowledge about work ability in occupational sub-groups is needed.

## Introduction

Musculoskeletal disorders (MSDs) are a major problem for patients as well as for society and can lead to functional limitation and absence from work [1, 2]. Health care workers have physically and psychologically demanding work and are at high risk of developing long term MSDs and sickness absence [3–5].

Research regarding work ability and prevention of sickness absence is a great challenge because of its complexity. The International Classification of Function (ICF) provides a classification system for function and disability associated with health. The theoretical model of ICF explains functioning as all body function, activity and participation as well as personal and environmental factors that interact with these concepts [6]. Hence, work (dis)ability may be explained by physical, mental and social aspects of functioning, in addition to environmental and organizational demands of a person’s work and personal factors that influence his or her capacity to meet these demands. These aspects have been investigated in a number of studies. Socio-demographic factors such as age, gender and educational level are important predictors for work ability [7–11]. Other factors associated with insufficient work ability are heavy physical work [9, 10, 12, 13], high pain intensity [12, 14], social and environmental workplace factors [15, 16], and psychological variables [8, 11, 17]. Besides these factors, some studies have focused on the relation between deconditioning and poor work ability [11, 18–20]. Deconditioning refers to a decrease of capacity over time expressed by weakened muscle strength, reduced aerobic fitness or altered coordination during activity. Although it is argued that deconditioning may be a result of fear avoidance and altered behavioral performance, the evidence is inconclusive [19, 21–24]. There is also conflicting evidence concerning deconditioning among patients with chronic low back pain (LBP) [19, 25, 26].

Although self-reported functioning and physical tests have been used to predict and evaluate work ability in several studies, only a few studies have compared the function of employees on sick leave and employees still working despite MSDs [17, 27–30]. It has been found that employees on sick leave have poorer health and more disability [17, 28], higher perceived workload [27], more fear-avoidance beliefs [27, 29], lower pain acceptance [17, 27, 28] and lower functional capacity [11] compared to employees still working. More knowledge about the differences between employees on sick leave due to MSDs and employees staying at work despite MSDs can give us insight into what could be emphasized in work interventions and contribute to increase work participation.

Employment policies in Scandinavian countries have focused on active approaches for employees with reduced work ability. Partial sick leave has been used to give employees the possibility to combine work and sickness benefits [31]. However, there is a lack of evidence regarding functional ability in workers on partial sick leave compared to those on full sick leave.

The aim of this study was to describe self-reported and physically tested function in health care workers with MSDs and to examine how function was associated with work participation. By using the ICF’s model to understand the complexity of work ability, a wide range of bio-psycho-social and work-related factors were investigated. This study examines possible differences of functioning in (a) health care workers staying at work despite MSDs, (b) on partial sick leave, or (c) on full sick leave.

## Methods

This study was part of a larger study called “Function, Activity and Work” of health care workers with MSDs in the Municipality of Bergen. A cross-sectional study was conducted.

### Participants

The participants were recruited from the Department of Health and Social Service in the Municipality of Bergen, Norway, from January 2012 to December 2013. About 7,000 health care workers are employed in this department; working in nursing homes, home care service and in special homes for disabled. Through their managers and/or brochures we invited employees who were on sick leave or at risk of being sick-listed due to MSDs, to a functional evaluation. Health care workers with MSDs took direct contact with the University of Bergen and booked an appointment with a physiotherapist in the project. Exclusion criteria were insufficient knowledge of the Norwegian language and being on full sick leave for more than 4 months continuously.

### Procedure

Within 2 weeks after requesting an appointment, the participants met for an evaluation completed by a physiotherapist in the project. First, the participants filled in personal background data and standardized questionnaires. This took about 30 minutes. Thereafter they were examined by standardized physical tests for 15–20 minutes. The evaluation ended with a verbal and written presentation of the self-reported and physical findings for all participants, except 56 who were recruited to an randomized controlled trial for participants with low back pain (LBP).

The testers were two experienced physiotherapists who were familiar with the tests. They practiced several times together before the start of the project and also examined the first 10 participants together. The participants performed the tests that required minimal effort first, in order to prevent fatigue and pain from having a significant impact on scores.

### Measures

#### Self-Report

Age, gender, marital status, number of children, education, exercise, smoking, and duration of sick leave were registered. In addition, different questionnaires regarding pain, function, psychosocial health and work environment were filled in. For the logistic regression analysis we dichotomized educational level into secondary school/vocational education and university degree, episodes of sick leave into 0–1× and ≥2×, exercises into <1×/week and ≥1×/week, and smoking to yes (yes, daily and yes, sometimes) and no.

Pain intensity was assessed by Numeric Pain Rating Scale (NPRS) [32]. The NPRS has shown better reliability and responsiveness than the visual analogue scale [33, 34].

The participants marked on a pain drawing the area or areas that had been painful the last 14 days [35].

Subjective Health Complaints inventory (SHC) consists of 29 items regarding subjective somatic or psychological complaints experienced during the last month [36]. The SHC inventory has shown satisfactory test–retest reliability in students and patients with LBP [37].

Hopkins Symptoms Checklist (HSCL-25) has 25 items with 10 items for anxiety symptoms and 15 for depression symptoms [38]. The HSCL has been shown to have a satisfactory validity and reliability in psychiatric outpatients and in a normal population [38, 39].

The Tampa Scale of Kinesiophobia (TSK) [40] consists in short form of 13 items concerning fear of movement/re-injury. The TSK has been validated in numerous studies including patients with neck pain, acute and chronic LBP and fibromyalgia [41–43].

Örebro questionnaire predicts risk for future work disability. The short form of the Örebro Musculoskeletal Pain Screening Questionnaire has 10 items and is appropriate for clinical and research purposes since it is nearly as accurate as the longer version [44].

Norwegian Function Assessment Scale (NFAS) is an instrument for self-report of work related functioning with basis in the ICF’s classification system [45]. Test–retest reliability has been tested in a normal population and found acceptable [45].

To measure social and psycho-social characteristics of jobs the Demand-Control-Support Questionnaire (DCSQ) was developed by Theorell et al., based on a shortened and modified version of the Job-Demand- Social Support Model (JCQ) [46, 47]. The psychometric properties of DCSQ have been demonstrated to be satisfactory [46, 47].

The Short Form-12 (SF-12), a 12-item version of the SF-36, was used to measure physical and mental health-related quality of life [48]. The SF-12 has shown good internal consistency, validity, and responsiveness in patients with LBP [49].

Sleep disturbance was measured by the Bergen Insomnia Scale (BIS) [50]. BIS can refer to high internal consistency, adequate reliability and good convergent and discriminative validity [50].

Body Mass Index was calculated by dividing weight (kg) by the square of height (m).

#### Physical Tests

The physical tests were chosen to get a general impression of physical function according to body functions or activities in the ICF’s model. A more detailed description of the tests is given in Table 1.

**Table 1:** Description of physical tests

Physical tests	Content	Score	ICF-dimensions
Global Body Examination (GBE) (51, 52)	Six tests: truncal flexibility and ability to relax during passive movements: Elbow-drop flexibility, lumbar-sacral flexibility, head rotation resistance and resistance to hip circumduction, hip-knee flexion and arm/shoulder flexion	Each test: 0–7. Total score for Flexibility: 0–42, higher score indicating reduced flexibility. Healthy (34 individuals): Median = 5.5, mean = 7.2	Body function
Back Performance Scale (BPS) (53, 54)	Five tests reflecting mobility-related activities for trunk and lower extremities (sock-test, pick-up test, roll-up test, fingertip-to-floor and a lift test where a box weighing 4 kg (women) or 5 kg (men) is lifted from floor to waist for 1 min).	Each test: 0–3. Total score: 0–15 with higher scores indicating worse function. Normative data for people without back pain (n = 150): Median = 0, mean = 0.8	Activity/participation
High lift test	A high lift test was a modified lift test included in BPS. The participants lift a box of 2 kg (for women) or 3 kg (for men) from waist to shoulder height and back again. The lifting technique was optional.	Number of lifts performed in 1 min is counted.	Activity/participation
Biering–Sørensen test (55–58)	Static endurance of the back. Participants are positioned prone with the upper body extending beyond the edge of the plinth and the lower body is fixed to the bench with three straps.	The length of time holding the upper body straight is recorded. Max time 240 s. Healthy (31 individuals): Median = 138	Body function
Abdominal endurance/strength (59, 60)	Three levels of dynamic sit-up test with increased demand for each level. The participants are supine with the knees flexed and with feet supported on the plinth by the tester.	The number of completed repetitions is counted (0–15).	Body function
Tender points (61)	18 defined fibromyalgia tender points with four kilos pressure are tested.	Painful points are counted	Body function

The Global Body Examination (GBE) is used to assess bodily function in patients with long-lasting musculoskeletal pain and/or with psychosomatic complaints. Six tests of truncal flexibility and ability to relax during passive movements were chosen. Discriminating ability between healthy and different patients groups has shown to be very good to excellent [51]. Good inter-tester reliability has been demonstrated in a former version of the GBE [52].

Back Performance Scale (BPS) consists of five tests reflecting mobility-related daily activities for trunk and lower extremities. Satisfactory test–retest reliability and responsiveness to change have been demonstrated in patients with long-lasting LBP [53, 54].

A high lift test was also performed. This is a modified lift test from the lifting test in the BPS, but not described elsewhere.

To assess static endurance of the back extensors we used the Biering–Sørensen test [55]. Test–retest reliability has been reported as satisfactory, but variability has been high [56–58].

For testing of abdominal endurance/strength we chose a three levels dynamic sit-up test with increasing demands for each level [59, 60].

We also included testing of tender points to get an impression of widespread pain [61]. Four kilos pressure of 18 defined fibromyalgia tender points were tested, and painful points counted.

The study was accepted by the Regional Committee for Medical and Health Research Ethics, Western-Norway, and was performed according to the Helsinki Declaration. Each participant signed an informed consent form prior to the examination.

### Statistical Analyses

Statistical analyses were calculated using SPSS (version 19; SPSS Inc., Chicago, IL, 2011) and Matlab (version 7.10; MathWork, 2010).

Descriptive statistics were used for demographic variables for all participants. Several variables were not normally distributed and non-parametric tests were therefore used. Differences between groups (full sick leave, partial sick leave, not sick leave/working) were analyzed by Chi square exact for categorical data and Kruskal–Wallis and Mann–Whitney U tests for continuous variables. A personal mean was given for missing data if <30 % of a sub-scale was missing.

To examine which factors were associated with being on sick leave, a logistic regression analysis was performed using sick leave groups as the dependent variable and several independent variables (gender, age, self-reported physical and mental function, perception of work environment, physical tests). We estimated both an unadjusted model for each independent variable and a fully adjusted model containing all independent variables. From those models and a correlation analysis we selected a final model based on statistical significance and clinical relevance. Some of the variables were dichotomized, as described in the method section. Work demands were reported in both back-ground data and in the DCSQ and reflect similar aspect. We chose the DCSQ in the logistic regression model because this is a standardized measurement tool. The general significance level was set to *p* < 0.05. Taking into account multiple effects, a Bonferroni adjustment was too conservative, therefore we used *p* ≤ 0.01 as marginal level.

## Results

A total of 250 participants (92.4 % women) were consecutively recruited to the functional evaluation study. Self-reports showed that 83 % of the participants had experienced their present complaints for more than 8 weeks. About 50 % reported previous contact with health personal for treatment of their MSDs. However, during their current episode the majority did not report any treatment. The group not on sick leave (working group) included 168 participants and the groups on partial and full sick leave each included 41 participants. In Table 2, demographic characteristics of the participants are provided. There were only women on partial sick leave. Workers on partial sick leave had statistically significant longer duration of sick leave compared to workers on full sick leave. The group on full sick leave reported more heavy physical work compared to the working group. The differences in function, health and work related variables between the three groups are presented in Table 3. Major differences in self-reported and physically tested function were observed between the group on full sick leave and the working group. Participants on full sick leave had statistically significant poorer function and higher (worse) score on Örebro questionnaire compared to those working. When comparing those on partial and full sick leave, the group on partial sick leave had statistically significant (*p* < 0.05) better scores on NFAS, the physical dimension of SF-12, NPRS, Örebro questionnaire, BSI, GBE and high lift test, compared to the group on full sick leave.

**Table 2:** Demographic variables

Variables	Gr.1 working N = 168 N (%)	Gr.2 partial sick leave N = 41 N (%)	Gr.3 full sick leave N = 41 N (%)	*p* value
*Sosiodemographic factors*				
Age^a^	49 (21–64)	47 (26–62)	49 (21–67)	.414^b^
Gender, women	155 (85.4)	41 (100)	35 (95.8)	.052^c^
Education				.273^c^
Secondary school	11 (6.6)	2 (4.9)	5 (12.2)	
Vocational education	82 (49.1)	23 (56.1)	24 (58.5)	
University degree	74 (44.3)	16 (39.0)	12 (29.3)	
*Work status*				
Full-time work	110 (65.5)	23 (56.1)	24 (58.5)	.436^b^
Sick leave (weeks)^a^	0 (0)	9 (2–62)	3 (0–10)	**<.001** ^**b**^
Sick leave episodes (number)				.214^c^
0×	27 (17.1)	8 (19.5)	10 (24.4)	
1×	32 (20.3)	13 (31.7)	10 (24.4)	
≥2×	99 (62.7)	20 (48.8)	21 (51.2)	
Type of work (mainly)				**.034** ^**c**^
Sedentary work/sitting	13 (7.8)	1 (2.5)	0 (0)	
Standing/walking	97 (58.1)	23 (57.5)	17 (42.5)	
Heavy physical work	57 (34.1)	16 (40.0)	23 (57.5)	
*Health related factors*				
Main disorder				.067^c^
Neck- and shoulder pain	53 (32.3)	14 (34.1)	7 (17.1)	
Low back pain	61 (36.3)	19 (46.3)	19 (46.3)	
Widespread pain	3 (25.6)	3 (7.3)	10 (24.4)	
Other	10 (6.0)	5 (12.2)	5 (12.2)	
Smoking				.403^c^
Yes, daily	31 (18.8)	9 (22.0)	13 (32.5)	
Yes, sometimes	18 (10.9)	4 (9.8)	5 (12.5)	
No	116 (70.3)	28 (68.3)	22 (55.0)	
Exercise				.752^c^
<1×/week	30 (17.9)	7 (17.0)	9 (21.9)	
1–2×/week	73 (43.5)	17 (41.5)	13 (31.7)	
3–5×/week	65 (38.7)	17 (41.5)	19 (46.4)	
Body mass index^a^	24.9 (18.8–42.1)	25.2 (17.6–39.6)	25.4 (17.2–36.4)	.904^b^

^a^Median (min–max)

^b^Kruskal–Wallis test

^c^Chi square, exact test Bold = significant at *p* < 0.05

**Table 3:** Differences in health, work characteristics and function between three groups: those working, on partial sick leave, or on full sick leave

Variables	N	Gr. 1 working	Gr. 2 on partial sick leave	Gr.3 on full sick leave	Kruskal–Wallis test
		Median (min–max)	Median (min–max)	Median (min–max)	*p* values
*Pain*					
Pain intensity	250	6 (2–10)	5 (3–10)	7 (2–10)	**0.005**
Pain drawing area	250	10 (1–70)	9 (2–37)	10 (1–40)	0.72
*Health factors and function*					
Ørebro questionnaire	250	44 (14–84)	46 (17–70)	56 (32–80)	**0.001**
SF-12 mental	232	50.1 (26.7–61.1)	48.6 (30.5–63.2)	48.4 (2.9–61.2)	0.412
SF-12 physical	232	45.5 (12.8–59.9)	42.2 (24.3–3.2)	38.7 (24.6–48.4)	**<0.001**
NFAS	250	1.2 (1.00–2.10)	1.23 (1 (1.00– 1.84)	1.42 (1.0– 2.38)	**<0.001**
HSCL	244	1.44 (1.00–2.87)	1.42 (1.00–2.58)	1.45 (1.04–3.08)	0.665
SHC (n)	179	10 (3–15)	9 (3–13.0)	10 (3–15)	0.245
TSK	247	21.7 (13.0–46.0)	21.0 (13.0–35.8)	21.0 (13.0–43.0)	0.952
BIS	244	16.5 (0–42)	17.0 (0–36)	24.0 (2–41)	0.065
*Work characteristics*					
DCSQ social	247	0.78 (0.22–1.00)	0.78 (0.33–1.00)	0.72 (0.33–1.00)	0.108
DCSQ demand	246	0.67 (0.00–1.00)	0.67 (0.27–0.93)	0.67 (0.27–1.00)	0.214
DCSQ control	240	0.67 (0.22–0.94)	0.64 (0.39–0.83)	0.67 (0.39–0.83)	0.186
*Physical assessment*					
ACR-tender points (n)	250	7 (0–18)	6 (0–18)	7 (0–18)	0.616
GBE flexibility	250	16 (2–35)	16 (5–30)	19 (5–35)	**0.038**
High lift test (n)	250	16 (0–29)	15 (8–24)	13 (3–25)	**<0.001**
Abdominal strength (n)	248	12.5 (0–15)	9 (0–15)	5 (0–15)	**<0.001**
Back strength (s)	248	70 (0–240)	33 (0–220)	36 (0–240)	**0.002**
BPS	250	3 (0–15)	4 (0–11)	6 (0–13)	**<0.001**

*SF-12* Quality of Life, Short Form-12, *NFAS* Norwegian Function Assessment Scale, *HSCL* Hopkins Symptoms Checklist, *SHC* Subjective Health Complaints, *TSK* Tampa Scale of Kinesiophobia, *BIS* Bergen Insomnia Scale, *BMI* Body Mass Index, *DCSQ* Demand-Control-Support Questionnaire, *ACR-Tender Points* American Criteria of Rheumatology, *GBE* Global Body Examination, *BPS* Back Performance Scale. Bold = significant at *p* < 0.05

The results of the logistic regression analysis are presented in Table 4. The group on full sick leave and the group on partial sick leave were compared with the working group. Complete data were available in 210 participants (142 working, 30 on full sick leave, 38 on partial sick leave). Reduced level of the physical dimension of SF-12 and on high lift test were significantly related to full sick leave (OR 0.86, *p* < 0.001) (OR 0.79, *p* = 0.002). There was also a tendency (*p* < 0.05) that being on full sick leave was associated with gender, the mental dimension of SF-12, the HSCL-25, the demand dimension of the DCSQ, and the abdominal strength test. The physical dimension of SF-12 (OR 0.91, *p* = 0.005) was the only variable that was associated to partial sick leave (Table 4). The full logistic regression model is shown in Table 5.

**Table 4:** Logistic regression comparing group on full sick leave and on partial sick leave with the working group

Variables	N	Unadjusted model, full sick leave			Adjusted model, full sick leave N = 30			N	Unadjusted model, partial sick leave			Adjusted model, partial sick leave N = 38		
		OR	95 % CI	*p v*alue	OR	95 % CI	*p v*alue		OR	95 % CI	*p v*alue	OR	95 % CI	*p v*alue
Gender	41	0.49	(0.17–1.38)	0.176	0.10	(0.02–0.67)	0.018	41			^a^			
Age	41	1.00	(0.97–1.04)	0.884	0.96	(0.92–1.01)	0.126	41	0.98	(0.95–1.01)	0.241	0.97	(0.94–1.01)	0.164
Smoking	40	0.67	(0.46–1.00)	0.049	0.84	(0.47–1.51)	0.567	41	0.92	(0.61–1.41)	0.711	1.16	(0.69–1.96)	0.580
SF-12 physical	37	0.86	(0.82–0.91)	**<0.001**	0.86	(0.79–0.94)	**<0.001**	40	0.91	(0.87–0.96)	**0.001**	0.91	(0.85–0.97)	**0.005**
SF-12 mental	37	0.96	(0.91–1.01)	0.110	0.89	(0.80–1.00)	0.043	40	0.98	(0.93–1.04)	0.509	0.95	(0.87–1.03)	0.210
HSCL	40	1.99	(0.84–4.72)	0.118	0.10	(0.01–0.90)	0.040	40	1.16	(0.45–2.98)	0.754	0.25	(0.04–1.43)	0.119
DCS support	41	0.12	(0.02–0.80)	0.029	0.67	(0.02–18.82)	0.816	41	1.20	(0.16–8.77)	0.861	1.96	(0.15–26.35)	0.611
DCS demand	40	3.63	(0.3–24.86)	0.189	37.07	(1.73–792.84)	. 021	41	4.75	(0.69–32.65)	0.113	6.65	(0.58–76.37)	0.128
DCS control	38	0.18	(0.01–2.88)	0.223	4.43	(0.03–578.40)	0.549	40	0.21	(0.01–3.34)	0.272	0.16	(0.01–4.88)	0.295
GBE flexibility	41	1.07	(1.02–1.12)	**0.009**	1.02	(0.94–1.10)	0.676	41	1.00	(0.95–1.05)	0.957	0.98	(0.92–1.04)	0.559
High lift test	41	0.82	(0.75–0.90)	**<0.001**	0.79	(0.68–0.91)	**0.002**	40	0.97	(0.89–1.06)	0.478	1.04	(0.92–1.17)	0.567
Abdominal strength	40	0.84	(0.78–0.91)	**<0.001**	0.84	(0.73–0.97)	0.014	41	0.94	(0.87–1.00)	0.065	0.95	(0.85–1.06)	0.335
Back strength	41	0.99	(0.98–1.00)	0.013	1.00	(0.00–1.01)	0.385	41	0.99	(0.99–1.00)	0.039	0.99	(0.99–1.00)	0.305
BPS	41	1.29	(1.15–1.45)	**<0.001**	0.85	(0.66–1.09)	0.206	41	1.15	(1.03–1.29)	0.016	1.06	(0.89–1.28)	0.513

*SF*-*12* Quality of Life, Short Form-12, *HSCL* Hopkins Symptoms Checklist, *DCSQ* Demand-Control-Support Questionnaire, *GBE* Global Body Examination, *BPS* Back Performance Scale. Bold = significant at *p* ≤ 0.01

^a^Only women

**Table 5:** Logistic regression - full model. Comparing full sick leave and partial sick leave with the working group

N = 189	N	Unadjusted model, full sick leave			Adjusted model, full sick leave			N	Unadjusted model, partial sick leave			Adjusted model, partial sick leave		
		OR	(95 % CI)	*p* value	OR	(95 % CI)	*p* value		OR	(95 % CI)	*p* value	OR	(95 % CI)	*p* value
Gender	41	0.49	(0.17–1.38)	0.176	0.06	(0.00–0.70)	0.025	41						
Age	41	1.00	(0.97–1.04)	0.884	0.96	(0.91–1.02)	0.160	41	0.98	(0.95–1.01)	0.241	0.98	(0.93–1.02)	0.284
Education	41	0.52	(0.25–1.09)	0.083	0.67	(0.19–2.39)	0.541	41	0.80	(0.40–1.62)	0.541	0.49	(0.17–1.38)	0.177
Working (full/partly)	41	1.37	(0.68–2.75)	0.381	1.93	(0.52–7.17)	0.324	41	1.51	(0.75–3.03)	0.245	1.16	(0.40–3.40)	0.781
Sick leave episodes	41	0.63	(0.31–1.25)	0.184	0.42	(0.13–1.38)	0.153	41	0.57	(0.28–1.13)	0.109	0.53	(0.20–1.38)	0.190
Smoking	40	0.67	(0.46–1.00)	0.049	0.72	(0.36–1.44)	0.351	41	0.92	(0.61–1.41)	0.711	1.25	(0.67–2.32)	0.486
Exercise	41	0.77	(0.33–1.79)	0.547	0.94	(0.19–4.70)	0.937	41	1.06	(0.43–2.61)	0.906	1.49	(0.34–6.45)	0.594
Pain categories	41	0.71	(0.47–1.08)	0.107	0.83	(0.33–2.09)	0.692	41	0.79	(0.52–1.19)	0.266	0.89	(0.46–1.73)	0.738
Pain drawing area	41	0.99	(0.96–1.02)	0.582	0.98	(0.93–1.04)	0.572	41	0.98	(0.94–1.02)	0.293	0.97	(0.92–1.02)	0.280
NPRS	41	1.33	(1.10–1.60)	**0.003**	0.95	(0.64–1.41)	0.809	41	0.95	(0.79–1.13)	0.546	0.77	(0.56–1.06)	0.112
Örebro questionnaire	41	1.05	(1.02–1.08)	**<0.001**	1.04	(0.98–1.11)	0.211	41	1.01	(0.99–1.04)	0.326	1.02	(0.96–1.08)	0.470
NFAS	41	20.89	(5.62–77.70)	**<0.001**	2.47	(0.13–48.00)	0.550	41	2.99	(0.72–12.47)	0.132	0.56	(0.03–9.48)	0.685
SF-12 physical	37	0.86	(0.82–0.91)	**<0.001**	0.88	(0.79–0.98)	0.015	40	0.91	(0.87–0.96)	**0.001**	0.89	(0.81–0.97)	**0.006**
SF-12 mental	37	0.96	(0.91–1.01)	0.110	0.91	(0.80–1.03)	0.131	40	0.98	(0.93–1.04)	0.509	0.94	(0.84–1.05)	0.246
HSCL	40	1.99	(0.84–4.72)	0.118	0.08	(0.01–1.15)	0.063	40	1.16	(0.45–2.98)	0.754	0.66	(0.08–5.35)	0.699
TSK	41	1.00	(0.95–1.06)	0.863	0.86	(0.76–0.98)	0.019	41	0.99	(0.94–1.05)	0.780	0.95	(0.86–1.05)	0.333
DCS social	41	0.12	(0.02–0.80)	0.029	0.34	(0.01–16.50)	0.583	41	1.20	(0.16–8.77)	0.861	3.13	(0.13–73.77)	0.479
DCS demand	40	3.63	(0.53–24.86)	0.189	153.40	(2.77–8,508.64)	0.014	41	4.75	(0.69–32.65)	0.113	5.53	(0.27–112.31)	0.266
DCS control	38	0.18	(0.01–2.88)	0.223	1.11	(0.00–363.95)	0.973	40	0.21	(0.01–3.34)	0.272	0.14	(0.00–11.13)	0.379
BIS	40	1.04	(1.00–1.07)	. 027	1.00	(0.95–1.07)	0.873	41	0.99	(0.96–0.02)	0.533	0.95	(0.91–1.00)	0.069
BMI	39	1.01	(0.93–1.10)	0.829	0.96	(0.82–1.12)	0.582	37	1.01	(0.92–1.10)	0.884	0.93	(0.81–1.05)	0.241
GBE, flexibility	41	0.12	(0.02–0.80)	0.029	1.05	(0.96–1.14)	0.326	41	1.00	(0.951.05)	0.957	1.01	(0.93–1.09)	0.832
ACR-18 tender points	41	1.04	(0.97–1.11)	. 303	0.96	(0.83–1.11)	0.562	41	1.03	(0.96–1.11)	0.361	0.99	(0.88–1.11)	0.811
High lift test	41	3.63	(0.53–24.86)	0.189	0.79	(0.66–0.93)	**0.006**	40	0.97	(0.89–1.06)	0.478	1.08	(0.93–1.26)	0.312
Abdominal strength	40	0.18	(0.01–2.88)	. 223	0.87	(0.74–1.03)	0.097	41	0.94	(0.87–1.00)	0.065	0.94	(0.83–1.07)	0.343
Back strength	41	0.99	(0.98–1.00)	0.013	1.00	(0.99–1.02)	0.597	41	0.99	(0.99–1.00)	0.039	0.99	(0.98–1.01)	0.314
BPS	41	1.29	(1.15–1.45)	**<0.001**	0.87	(0.631.21)	0.415	41	1.15	(1.03–1.29)	0.016	1.10	(0.87–1.38)	0.432

*NPRS* Numeric Pain Rating Scale, *NFAS* Norwegian Function Assessment Scale, *SF*-*12* Quality of Life, Short Form-12, *HSCL* Hopkins Symptoms Checklist, *SHC* Subjective Health Complaints, *TSK* Tampa Scale of Kinesiophobia, *DCSQ* Demand-Control-Support Questionnaire, *BIS* Bergen Insomnia Scale, *BMI* Body Mass Index, *ACR*-*Tender Points* American Criteria of Rheumatology, *GBE* Global Body Examination, *BPS* Back Performance Scale. Bold = significant at *p* ≤ 0.01

## Discussion

In this study we found that workers on full sick leave had reduced self-reported and physically tested function compared to workers still working despite MSDs, as well as compared to those on partial sick leave. Lower physical function measured by the physical function score on SF-12 and the high lifting test were strongest associated with being on full sick leave. Being female, lower mental health score (worse) on SF-12, in addition to lower scores (better) on the HSCL-25, increased self-reported work demands (DCSQ) and lower abdominal strength showed a tendency to be associated with being on full sick leave. For the group on partial sick leave, only the physical function scale of SF-12 was associated with being on sick leave, those on sick leave having lower (worse) scores.

Our findings are supported by several studies, but there are also new and interesting findings. Low self-reported physical health and disability have been found to be associated with being on sick leave in patients with chronic LBP [62]. In a systematic review [63] of factors that promote staying at work with MSDs, an association was found between low perceived physical disability and staying at work. However, only a few studies have compared measures of physical tests/capacity between workers on sick leave and workers who continue working despite pain. Soer et al. [11] compared functional capacity between workers staying at work despite MSDs, workers on sick leave due to MSDs and a group of healthy workers. In accordance with our findings, they found that the two groups with MSDs had significantly lower functional capacity than the healthy group, with the lowest capacity observed in the group on sick leave. Other studies have shown that physical tests can predict return to work after being on sick leave. Cardiovascular fitness was identified as one of the strongest predictors for return to work in a Norwegian study [19]. In a systematic review [18], better results on physical tests, and especially the lifting test, appeared to be predictive of work participation for patients with MSDs. As our study was cross-sectional, prediction of work participation could not be estimated. Low lifting capacity was, however, strongly associated with being on full sick leave. An explanation may be that lifting captures several components such as gripping, holding, bending and lowering. In addition, lifting can be influenced by pain and fear of movement.

Several explanations were considered in order to explain why the participants on full sick leave in the present study had lower scores on the physical tests compared to workers not on sick leave. A possible explanation could have been different level of exercise between groups. However, the three groups in the present study reported quite similar level of regular exercising, in accordance with earlier research [12, 27]. Another aspect might be fear of pain and movement. Increased fear avoidance has been observed in workers on sick leave with MSDs [19, 27, 29]. Our findings did not support this association, as scores on the TSK were similar for those on sick leave versus those working.

Reduced physical function does not necessarily lead to limitation of work participation. Even if a state of deconditioning is present, the functional capacity could still be sufficient to meet actual work demands, especially if they are not too excessive [11]. However, health care workers usually have physically demanding work, including lifting, transferring patients and working in uncomfortable positions. In accordance with several studies showing that perceived workloads are associated with being on sick leave [12, 19, 27], the workers on full sick leave in this present study reported higher perceived work demands than the other two groups. The reason might be more demanding work tasks for this group, but decreased physical capacity might also influence an individual’s perception of work demands. This highlights the need for research that takes into account work demands and work environment for specific occupational groups.

High pain intensity has also been associated with being on sick leave [8, 11, 17]. Our study showed a statistically significant difference of pain intensity between the groups, with the highest level in the group on full sick leave and the lowest in the group on partial sick leave. However, there was only one point in difference on the NPRS between those on full sick and the working group. Only a few of the participants reported increased pain after the physical tests. It is therefore not likely that the pain level was of great importance for the result regarding physical functioning in the present study.

In previous years, much attention has been given to the role of psycho-social factors related to work ability [17, 64]. There were only small differences in measures of the psychological variables between the groups in our study. Reduced physical function was more strongly associated with being sick-listed than pscyho-social factors, also reported in previous research [12, 27]. There was only a tendency that being on full sick leave was associated with mental health, and the results were conflicting. The group on full sick leave showed worse function at the mental health component of SF-12, but surprisingly, better score on HSCL-25. The HSCL-25 has a higher number of items related to mental health and may therefore provide a more precise picture than the less detailed generic questionnaire SF-12. Being on short time sick leave, as in our study, may to a lesser degree influence psycho-social factors.

The authorities in Norway, Sweden and Denmark have strongly promoted the use of partial sick leave as the primary choice, if sick leave is needed. It is assumed that partial sick leave has positive effects on health and well-being, compared to full-time absence, and it is believed to facilitate return to full-time work [31]. To our knowledge, the present study is the first study comparing self-reported and physical tested function in workers with those on full or partial sick leave, due to MSDs. The group on partial sick leave had statistically significant better function on some of the functional questionnaires and physical tests compared to those on full sick leave. Interestingly, there were only women in the partial sick leave group. More women than men have been on partial sick leave according to register data from Norway [2]. Further research is needed to get insight into factors affecting workers on partial and full sick leave, and the decisions around sick leave.

### Strengths and Limitations

The high number of participants in our study (n = 250) gave us enough power to detect differences between workers on full, partial or not on sick leave, and to identify variables related to work status. In accordance with the ICF- model [6] a variety of demographic variables, questionnaires and physical tests were used to cover the different dimensions in the model when evaluating the participants’ functioning and working ability. We used well-known standardized questionnaires measuring pain, physical- and mental functioning and conditions at work. In addition, we used standardized physical tests. This is in line with Wand et al. [65] who argued that both self-reported and physically tested functioning need to be assessed to get a better understanding of MSDs and how they could be managed. The physical tests we used were likely to reflect function in different MSDs. The testing was well tolerated by the participants. The tests demonstrate good levels of reliability and validity, but two of the tests (abdominal and high lift tests) are still under evaluation. The physical tests were able to discriminate functioning between workers on sick leave and not, although most of the workers were not on long-term sick leave. This indicates that the test battery could be a useful assessment of function at an early stage of sick leave and a tool when giving advice about rehabilitation and work adaption. Different batteries of physical tests are designed to evaluate work ability and daily functioning [18, 66, 67]. Most of them are costly and time-consuming and are mainly used as assessment tools in the return to work process. In contrast to this, our test battery is cheap, quick to apply and require little equipment and therefore could also be a useful clinical tool in private practice for physiotherapists.

Workers were provided with information about the project by their leaders and through pamphlets and took direct contact to participate. A threat to the external validity is a possible selection bias. Although several workers were “pushed” by their employer to participate, we cannot be sure that the least motivated and the workers with more complex health problems actually contacted us. Our target population was workers on sick leave or at risk of becoming sick-listed due to MSDs. Only 20 % of all that were examined had never been on sick leave due to MSDs before; indicating that we have included the target group. Interviews with managers in the midst of the total project supported that we had managed to get a representative sample of participants (not yet published).

The present study only included self-reported data on sick leave. Although self-reported sick leave data has been evaluated as being less reliable than register recorded data [68], other studies [69, 70] have demonstrated good agreement between self-reports and register data in cross-sectional design. The workers’ sick leave history is only partial known. The length of the last sick leave and number of sick leave episodes the last years are reported, but not the length of all absences. There could also have been changes in job status in the period before assessment. Workers on sick leave could recently have returned to work, and workers on partial sick leave could have changed to full sick leave. Our study did not record this, and it is quite surprising that the differences between the groups still were so significant.

Over 90 % of the participants in the present study were women working in the health- and social sector. This limits the generalizability of the study. Being male and/or having a less demanding work may not affect work ability in the same way.

The present study was cross-sectional and therefore causality cannot be inferred, and only associations are reported. It was conducted in a single country with a highly established social insurance system, thereby reducing generalizability of the study to countries that have similar social and security system.

More specific knowledge about occupational sub-groups is needed to catch groups at risk for prolonged sick leave, and further research in this field should emphasize longitudinal studies.

## Conclusion

Health care workers on full sick leave due to MSDs have reduced function on self-reported and physically tested function, compared to those working despite MSDs, as well as compared to those on partial sick leave. Lower physical function measured by the physical dimension on SF-12 and the high lift test were strongest associated with being on full sick leave, and only the physical dimension on SF-12 was associated with being on partial sick leave. More knowledge about work ability in occupational sub-groups is needed.
